# Comparison of Feng spinal mobilization with Maitland mobilization in management of chronic nonspecific low back pain: A cohort study

**DOI:** 10.1097/MD.0000000000032984

**Published:** 2023-02-17

**Authors:** Cheng Gong, Shiyin Dai, Bing Jin, Ying Xie

**Affiliations:** a Department of Rehabilitation Medicine, Beijing Friendship Hospital, Capital Medical University, Beijing, China.

**Keywords:** chronic nonspecific low back pain, lumbar range of motion, mobilization, pain

## Abstract

Feng spinal mobilization (FSM) is one of the most widely practiced techniques in traditional Chinese osteopathy, especially in China. However, whether this FSM technique is more effective than the Maitland posteroanterior mobilization (MM), which is widely used all over the world, is still unknown. The purpose of this study was to retrospectively analyze and compare the efficacy of these 2 treatments in patients with chronic nonspecific low back pain (CNLBP) as to provide a basis for the clinical treatment of chronic low back pain. A total of 83 patients, including 43 patients in the FSM group and 40 in the MM group, were enrolled in this cohort study. FSM or MM was performed on patients 3 times during a period of 2 weeks. Changes in the subjective and objective measurements were measured before and after the third treatment. The subjective symptoms recorded included the visual analogue scale (VAS), Oswestry disability index, and Patient Health Questionnaire-9. The objective symptoms, including the lumbar range of motion (ROM), and straight leg raise (SLR) height were also checked for any changes. The VAS scores were reassessed at the 1-year follow-up visit. The results showed that 2 weeks of FSM treatment significantly improved CNLBP patients modified Schober test (*P* < .05), extension ROM (*P* < .01), and SLR height (*P* < .05) while MM treatment did not. Both treatments significantly decreased the values of VAS, Oswestry disability index, and Patient Health Questionnaire-9 (*P* < .01). Compared to the MM treatment, the FSM treatment showed a much more significant improvement in VAS score (*P* < .01), range of motion of extension (*P* < .01), and SLR of both sides (*P* < .05). At the 1-year follow-up, VAS scores in both groups decreased significantly compared to pretreatments; however, there was no significant difference between the 2 groups. Our data suggested that the FSM treatment can provide better efficacy than MM in CNLBP patients, improving the VAS scores, lumbar extension ROM, and SLR height in a shorter time.

## 1. Introduction

Low back pain is one of the most prevalent diseases of the skeletal muscle system in the adult population worldwide.^[1]^ Chronic nonspecific low back pain (CNLBP) is defined as lumbar pain persisting for longer than 3 months with no suspected pathology.^[1]^ It is reported that up to 84% of people have experienced low back pain at some time in their life, and the incidence of CNLBP reached 23%.^[2]^ In addition, CNLBP has many adverse effects on patients, including pain, dysfunction, and even disability which can seriously affect patients quality of life and social participation.^[3]^

Compared to the defined pathology of LBP, the pathologies of CNLBP are relatively less remarkable,^[4]^ which might be due to complex interactions among the biological, psychological, and social factors.^[5]^ It is proven that one of the key pathomechanisms of CNLBP is sensitization of the nervous system.^[5]^ Structural and functional changes in the brain have also been identified in CNLBP patients.^[5]^ Studies have shown that white and gray matter regions, such as the dorsolateral prefrontal cortex, thalamus, temporal lobes, insula, primary somatosensory cortex, change in the brain of CNLBP patients, and suggesting that CNLBP may have an association with the structural reorganization of the brain.^[6]^ Besides, alterations in the blood flow and metabolism also indicate changes in brain functions.^[7]^

Spinal mobilization, a frequently used conservative manual technique for CNLBP, is defined as low-velocity, nonthrusting, and passive movement within or at the limit of the joint range of motion.^[8]^ In recent guidelines and reviews, evidence has confirmed mobilizations as an effective treatment and has had a positive effect.^[9]^ Notably, all manual techniques have more pronounced neurophysiological and less mechanical effects than previously thought.^[10]^ In fact, mobilization techniques can promote sympathoexcitation, decrease mechanical hypoalgesia, and normalize muscle activity via the central pathway.^[11]^ It can also stimulate the myelinated alpha-beta and alpha-delta fibers to transmit impulses faster, thereby inhibiting the pain symptoms via altered signal transmission through C fibers. Additionally, mobilization can inhibit pain by increasing endorphin secretion.^[12]^ In summary, the neurophysiological and biomechanical mechanisms and the interaction between them can explain the reason for the effectiveness of the spinal mobilization despite its implementation heterogeneity.^[13]^

Among all the mobilization techniques, Feng spinal mobilization (FSM), Maitland posteroanterior mobilization (MM) are widely used techniques in China and around the world.

FSM is traditional Chinese osteopathy that is still popular in China today. It was created by professor Feng Tianyou in the 1970s.^[14]^ The FSM technique is performed when the patient is in a seated position. One of the therapist hands holds the patient superior shoulder to stabilize and rotate the lumbar spine, while the therapist thumb of the other hand applies gentle pressure to the specific spinous process and correct its deviation so as to relieve the postarticular joint capture. No cracking sound is produced during this technique.^[14]^ This technique has the advantage of quick response and has been proven effective for people with low back pain and leg pain due to lumbar disk herniation.^[15]^ However, evidence of its effectiveness on CNLBP is still insufficient.^[16]^ According to the theory of Professor Feng, the founder of FMS, this technique can correct the minor positional faults of the vertebrae and joints, which lead to neuromuscular disorders.^[16]^ Recently, FSM has been shown to alleviate pain by regulating the TRPV4/NO pathway in the dorsal root ganglion neurons, indicating the neurophysiological involvement in the FSM mechanisms.^[17]^

MM has been widely recognized all over the world.^[18]^ It is performed when the patient is in a prone position. Passive and posterior-anterior oscillation forces are given by the therapist to the affected spinal vertebrae to improve neuromuscular pain and stiffness.^[19]^ The MM regulates the neurophysiological mechanisms.^[11]^ It has been revealed that nonsegmental hypoalgesia and sympathoexcitation appear after the MM treatment, indicating the involvement of the central nervous system in suppressing pain symptoms. And an increase in the nociceptive flexion reflex suggests alterations in the spinal cord excitability.^[11]^

Until now, there have been no studies comparing the efficacy of these 2 treatments for CNLBP, so which method has better short-term and long-term results is still unknown. Hence, this study aims to compare the effects of the FSM and MM on pain, back pain-related functional disabilities,^[18]^ psychological state, and range of motion in patients with CNLBP. Consequently, this study aims to provide evidence for the clinical treatment of CNLBP.

## 2. Materials and methods

### 2.1. Study design and study population

This is a retrospective cohort study. Between September 2020 and September 2021, outpatients diagnosed with CNLBP in the Beijing Friendship Hospital, Capital Medical University with the following eligibility criteria were studied^[20]^:

Males and females ages between 18 and 68 years old; Suffering from low back pain for more than 12 weeks; With or without leg pain above the knee; Pain on the visual analogue pain scale (VAS) ≥ 2/10; In clinical practice, patients randomly received FMS or MM, and they all received exercise therapy.

Patients with any of the following symptoms were excluded: spinal cord and nerve root compression irritation, spinal cord and nerve root tumors, cauda equina injury and other neurological pathology symptomatic period; spinal pathology including spinal tumors, fresh fractures of spinal vertebrae and accessories, severe osteoporosis, and spinal instability; cardiovascular diseases including severe hypertension, cardiovascular disease symptomatic period, and aortic aneurysm; metabolic diseases; recovery from larger surgical operations; acute infection or pregnancy. The study was approved by the institutional ethical committee of Beijing Friendship Hospital, Capital Medical University, Beijing, China (Ethics Number: 2022-P2-190-01). All participants signed the informed consent before the data collection.

### 2.2. Procedure

In clinical practice, patients randomly received either FMS or MM, and all of the patients received the same exercise therapy. That is, each treatment session consists of FMS and exercise, or MM and exercise. In addition, patients were treated once every 4 days for 40 minutes at each treatment session. Each patient received a total of 3 treatment sessions during a period of 2 weeks.

Measurements were taken and recorded before the first treatment and immediately after the third treatment. The outcome measures were assessed by another therapist who was not involved in treatment and was blind to which treatment the patient received. Telephone follow-ups were conducted on patients pain scores 1-year after the end of the third treatment.

### 2.3. Intervention

#### 2.3.1. Feng spinal mobilization.

**Figure 1.**
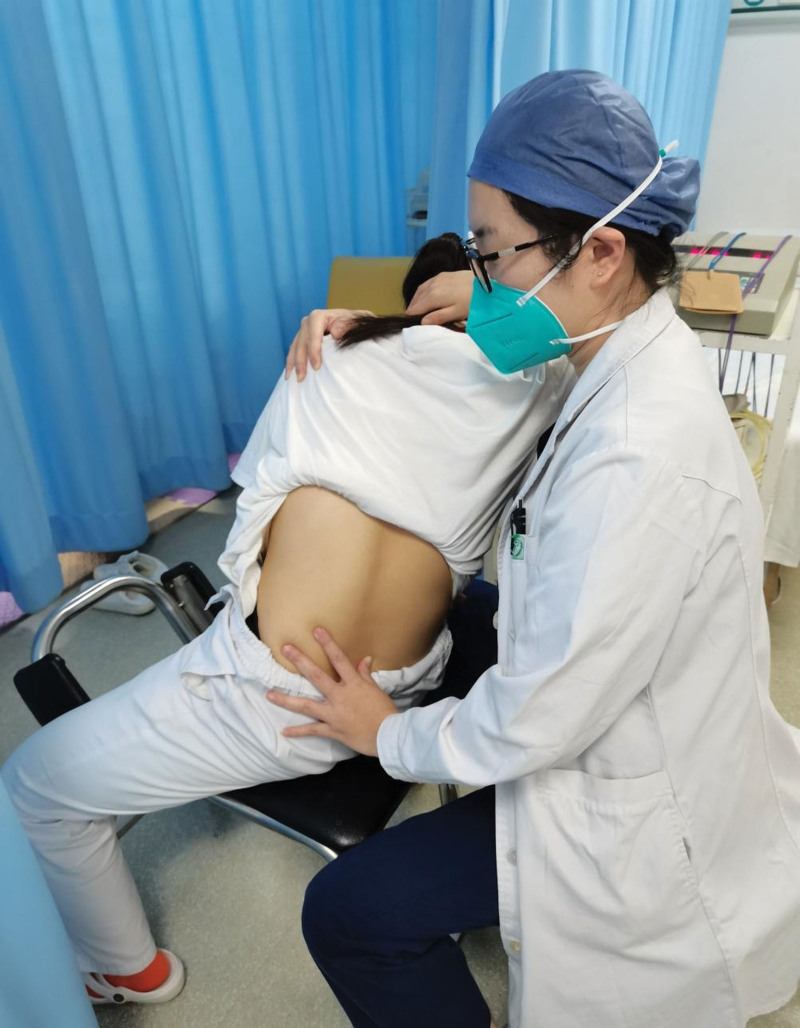
The Feng spinal mobilization approach.

The FSM approach is one of the most recognized spinal mobilization treatments in China.^[14]^ It was developed by Professor Feng Tianyou in the 1970s. This technique was performed on a deviated lumbar vertebra with a minor positional fault diagnosed by clinical examinations.^[16]^ Different from other mobilization used, this technique is conducted in a sitting position rather than a prone position. The therapist pushes against the spinous process of involved segments from behind with the thumb of 1 hand, and the other hand holds the opposite shoulder from the front of the body and slowly rotates the lumbar spine so as to get the apex of the rotational torque to reach the involved segments, then slowly returns the patient returned to the neutral position. The same procedure is repeated by rotating in the opposite direction to complete the mobilization (Fig. 1). The FMS is not a thrusting motion, so it does not induce cracking sounds in the joints.^[14]^ The therapist in this study had more than 5 years of clinical experience in manual physiotherapy and was skilled in performing FSM.

#### 2.3.2. Maitland mobilization.

This posteroanterior glide was performed on the involved lumbar vertebra, which was stiff, depending on the clinical findings of a therapist. The patients were in a prone position while the therapist applied a large amplitude of oscillatory posteroanterior forces to the end range of each involved segment. Each cycle lasted for about 60 seconds, for 3 cycles and 3 minutes in total.^[21]^ The therapist in this study had more than 5 years of clinical experience in manual physiotherapy and was skilled in operating MM.

#### 2.3.3. Exercise therapy.

Both groups received exercise therapy. The exercise consisted of general and specific exercises for the spine, pelvis, lower limbs. This therapy also included spinal flexibility exercises, static stretching, and core stability exercises^.[22–24]^ Spinal flexibility exercises consisted of camel-cat stretching with 2 to 3 sets, 10 to 15 reps per set. Static stretching exercises included stretching the paraspinal muscles, gluteus muscles, piriformis, and iliopsoas muscles, each for 15s, 3 to 5 sets. Core stability training included core breathing, transverse abdomen activation training, and bird-dogs and gluteus bridges at 2 to 3 sets, 10 to 15 reps per set. There was 30seconds to 1minute of rest between each set. The therapists instructed the patients to focus on the target and do the correct movements and told them not to provoke pain during the exercises.^[25]^ The exercise difficulty gradually advanced.

### 2.4. Outcome measures

#### 2.4.1. Visual analogue pain scale.

The VAS was used to assess the severity of pain when the patients were asked to rate their average level of pain.^[26]^ The VAS score ranges from 0 to 10 points. Zero point represents no pain, while 10 points represent extreme pain meaning the higher the score, the more severe the pain. The VAS score has demonstrated good reliability and validity in past studies.

#### 2.4.2. Oswestry disability index.

The Oswestry disability index (ODI) assesses the impacts of low back pain or leg pain on physical function and activities of daily living. There are 11 questions in the questionnaire, and the resulting score ranges from 0% to 100%; the higher the score, the more severe the disability.^[27]^

#### 2.4.3. Patient Health Questionnaire-9 (PHQ-9).

PHQ-9 is a widely used scale tool for screening, diagnosing, monitoring, and assessing the severity of depression.^[28]^ The PHQ-9 has 9 questions in total, representing 9 symptoms of depression. Each question is rated 0 to 3, the total score is added to equal 0 to 27. The higher the score, the more serious the depression state.

#### 2.4.4. Lumbar range of motion.

The lumbar range of motion (ROM) was measured by the modified Schober test (mSchober test) and finger-to-floor test.

The modified Schober test (mSchober test) includes a way to measure the active lumbar flexion ROM.^[29]^ Each patient stood with their feet positioned 8 cm apart on a box. A blinded assessor made 2 skin markers, including 5 cm below the lumbosacral junction and 10 cm above the lumbosacral junction. Then, each patient was instructed to actively bend forward as far as possible within the limit before pain and with the knees extended. The change of distance between the 2 skin markers was measured by a tape and recorded. According to other study references, this is an exceptionally reliable assessment for lumbar ROM.^[29]^

The fingertip-to-floor test was conducted to measure the patients ROM.^[30]^ The patient was instructed to stand on a box 20 cm high. First, a tape measure was used to measure the fingertips to the floor. Then the patient was asked to bend forward, reaching toward the floor, and the distance of the fingertips to the floor was measured again. The change in the distance is the flexion ROM of the patient. The extension and side bending ROM was measured in the same way.

#### 2.4.5. Straight leg raise (SLR).

The patient was in a supine position. The patient was asked to raise the lower limb actively, then the height of the lateral malleolus to the bed was measured with a tape measure. The SLR test can be restricted by the muscles or nerve tension.

### 2.5. Data analysis

Data analysis were done using SPSS 20.0 software (IBM Corporation, New York, NY). Data values were presented in mean ± S.D. The chi-square test was used to compare the sex of the 2 groups. The other demographic characteristics were compared between both groups by independent *t* test. Paired *t* tests were used for comparison within the group before and after the treatment. Any analysis with a *P* < .05 was considered statistically significant.

## 3. Result

### 3.1. Baseline characteristics of study patients

Eighty-three patients met the inclusion and exclusion criteria and participated in this study. Among all, 43 patients received the FSM approach, and 40 patients received the MM approach. The baseline information collected from both the FMS group and MM group is outlined in Table 1. There were no significant statistical differences between the 2 groups in baseline characteristics such as age, gender, weight, height, VAS, ODI, PHQ-9, lumbar range of motion, and SLR height (Table 1).

**Table 1:** Baseline characteristics of the chronic nonspecific low back pain patients in 2 groups.

	FSM group patients (n = 43)	MM group patients (n = 40)	*P* value
Gender (M/F)	18/25	17/23	.953
Age (yr-old)	37.05 ± 12.31	42.68 ± 15.47	.072
Height (cm)	168.70 ± 7.88	166.91 ± 7.48	.292
Weight (kg)	64.00 ± 11.95	67.70 ± 14.05	.193
VAS	4.47 ± 1.62	3.95 ± 1.84	.179
ODI (%)	26.26 ± 16.76	26.94 ± 14.76	.845
PHQ-9	6.30 ± 5.42	4.80 ± 5.26	.204
mSchober test (cm)	6.11 ± 2.77	5.77 ± 1.90	.520
Flexion ROM	46.39 ± 19.47	52.09 ± 15.40	.145
Extension ROM	5.57 ± 4.01	6.14 ± 3.83	.513
Left flexion ROM	16.94 ± 5.10	16.48 ± 6.86	.724
Right flexion ROM	17.17 ± 6.08	17.01 ± 7.40	.918
SLR – L (cm)	80.03 ± 7.41	81.86 ± 6.14	.227
SLR – R (cm)	80.09 ± 7.91	80.75 ± 6.10	.674

Values are presented as mean ± S.D. *P < .05, **P < .01 compared to pretreatment, #*P* < .05, ## *P* < .01 compared to MM treatment.

CNLBP = chronic nonspecific low back pain, FSM = Feng spinal mobilization, MM = Maitland posteroanterior mobilization, mSchober test = modified Schober test, ODI = Oswestry disability index, PHQ-9 = Patient Health Questionnaire-9, ROM = lumbar range of motion, SLR = straight leg raise, VAS = visual analogue pain scale.

### 3.2. Analysis of symptoms before and after FSM treatments over 2 weeks

**Figure 2.**
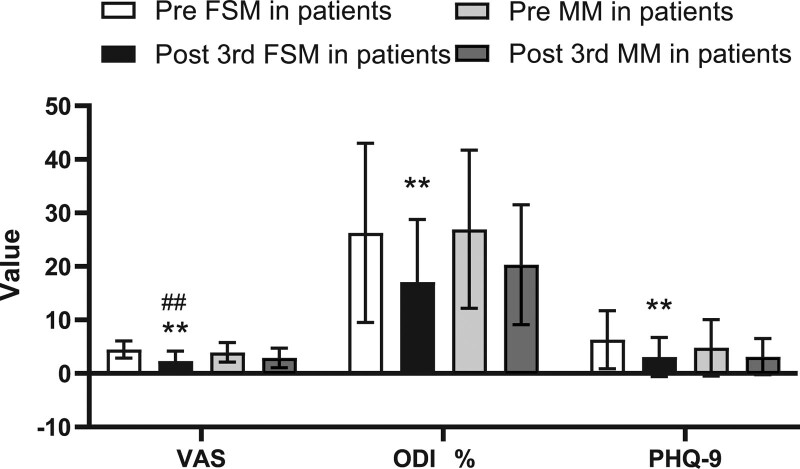
Comparison of subjective symptoms between 2 groups pre and post the 3rd treatment. FSM = Feng spinal mobilization, MM = Maitland posteroanterior mobilization, ODI = Oswestry disability index, PHQ-9 = Patient Health Questionnaire-9, VAS = visual analogue pain scale. Values are presented as mean S.D. *P < .05, **P < .01 compared to pretreatment, #*P* < .05, ## *P* < .01 compared to MM treatment.

**Figure 3.**
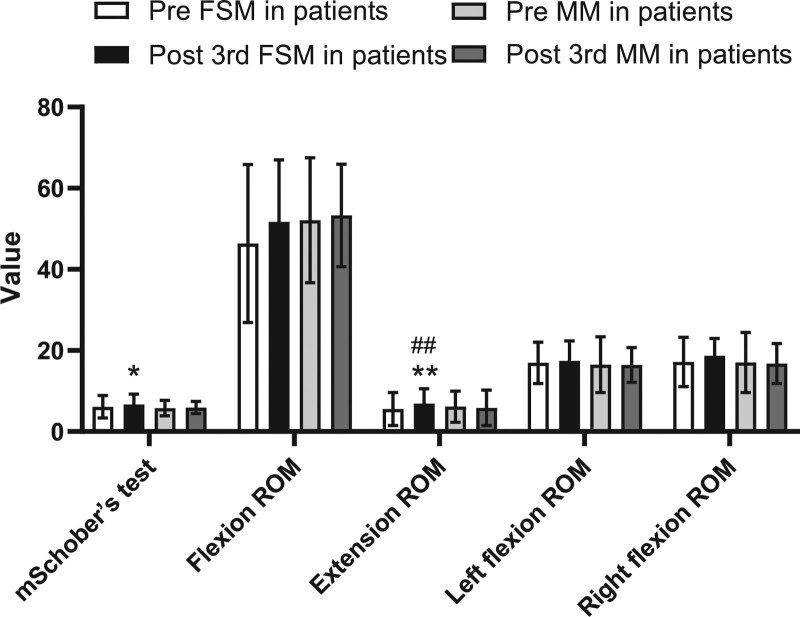
Comparison of range of motion between 2 groups pre and post the 3rd treatment. FSM = Feng spinal mobilization, MM = Maitland posteroanterior mobilization, mSchober test = modified Schober test, ROM = lumbar range of motion. Values are presented as mean S.D. *P < .05, **P < .01 compared to pretreatment, #*P* < .05, ## *P* < .01 compared to MM treatment.

**Figure 4.**
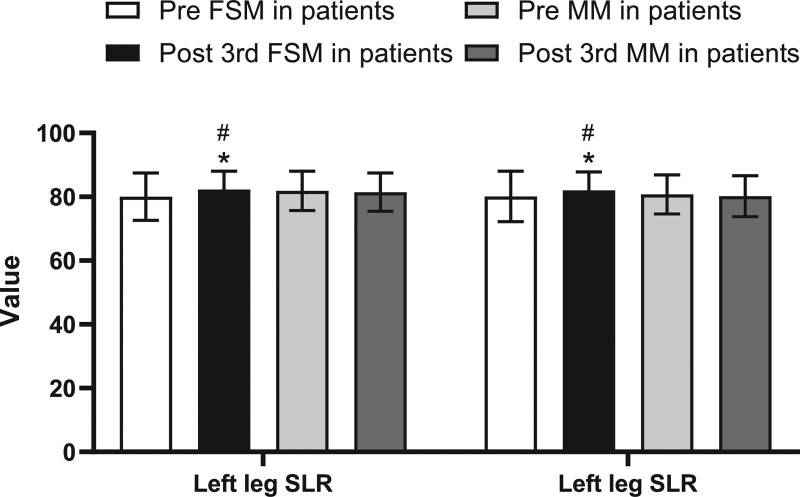
Comparison of straight leg raise height between 2 groups pre and post the 3rd treatment. FSM = Feng spinal mobilization, MM = Maitland posteroanterior mobilization, SLR = straight leg raise. Values are presented as mean S.D. *P < .05, **P < .01 compared to pretreatment, #*P* < .05, ## *P* < .01 compared to MM treatment.

After the FMS treatments, in 2 weeks the patients subjective symptoms were all improved: the average VAS score was significantly reduced from 4.47 ± 1.62 to 2.33 ± 1.87 (*P* < .01), the ODI value was significantly reduced from 26.26%±16.76% to 17.08%±11.70% (*P* < .01), and the PHQ-9 score was significantly reduced from 6.30 ± 5.42 to 3.07 ± 3.64 (*P* < .01) (Table 2, Fig. 2). Most of the objective outcome measurements were also significantly improved. The mSchober test result increased from 6.11 ± 2.77 to 6.70 ± 2.55 (*P* < .05), the extension ROM increased from 5.57 ± 4.06 to 6.87 ± 3.68 (*P* < .01), the left SLR height increased from 80.03 ± 7.41 to 82.30 ± 5.78 (*P* < .05), and the right SLR height increased from 80.09 ± 7.91 to 82.04 ± 5.79 (*P* < .05). Mild improvements in flexion ROM and side flexion ROM was observed after FMS treatment; however, the changes were not considered significant (*P* > .05) (Tables 3 and 4, Figs. 3 and 4).

**Table 2:** Comparison of subjective symptoms between 2 groups pre and post the 3rd treatment.

Outcome measures	Time	FSM group (n = 43)	MM group (n = 40)	*P* value
VAS	Pre	4.47 ± 1.62	3.95 ± 1.84	.179
	Post	2.33 ± 1.87**/##	2.90 ± 1.82**	.004
	*P* value	.0000001	.001	
ODI (%)	Pre	26.26 ± 16.76	26.94 ± 14.76	.845
	Post	17.08 ± 11.70**	20.31 ± 11.19**	.335
	*P* value	.000044	.000265	
PHQ-9	Pre	6.30 ± 5.42	4.80 ± 5.26	.204
	Post	3.07 ± 3.64**	3.13 ± 3.37**	.076
	*P* value	.000004	.010	

Values are presented as mean ± S.D. *P < .05, **P < .01 compared to pretreatment, #*P* < .05, ## *P* < .01 compared to MM treatment.

FSM = Feng spinal mobilization, MM = Maitland posteroanterior mobilization, ODI = Oswestry disability index, PHQ-9 = Patient Health Questionnaire-9, VAS = visual analogue pain scale.

**Table 3:** Comparison of range of motion between 2 groups pre and post the 3rd treatment.

Outcome measures	Time	FSM group (n = 43)	MM group (n = 40)	*P* value
mSchober test (cm)	Pre	6.11 ± 2.77	5.77 ± 1.90	.520
	Post	6.70 ± 2.55*	5.93 ± 1.51	.259
	*P* value	.038	.563	
ROM				
Flexion (cm)	Pre	46.39 ± 19.47	52.09 ± 15.40	.145
	Post	51.68 ± 15.31	53.29 ± 12.64	.284
	*P* value	.144	.364	
Extension (cm)	Pre	5.57 ± 4.06	6.14 ± 3.83	.277
	Post	6.87 ± 3.68**/##	5.85 ± 4.35	.002
	*P* value	.008	.074	
Left Flexion (cm)	Pre	16.94 ± 5.09	16.48 ± 6.86	.724
	Post	17.44 ± 4.92	16.43 ± 4.28	.634
	*P* value	.508	.959	
Right Flexion (cm)	Pre	17.17 ± 6.08	17.01 ± 7.40	.918
	Post	18.67 ± 4.32	16.79 ± 4.93	.200
	*P* value	.057	.841	

Values are presented as mean ± S.D. *P < .05, **P < .01 compared to pretreatment, #*P* < .05, ## *P* < .01 compared to MM treatment.

FSM = Feng spinal mobilization, MM = Maitland posteroanterior mobilization, mSchober test = modified Schober test, ROM = lumbar range of motion.

**Table 4:** Comparison of straight leg raise height between 2 groups pre and post the 3rd treatment.

Outcome measures	Time	FSM group (n = 43)	MM group (n = 40)	*P* value
Left leg SLR (cm)	Pre	80.03 ± 7.41	81.86 ± 6.14	.227
	Post	82.30 ± 5.78*/#	81.47 ± 5.99	.041
	*P* value	.038	.632	
Right leg SLR (cm)	Pre	80.09 ± 7.91	80.75 ± 6.10	.674
	Post	82.04 ± 5.79*/#	80.20 ± 6.43	.027
	*P* value	.041	.424	

Values are presented as mean ± S.D. *P < .05, **P < .01 compared to pretreatment, #*P* < .05, ## *P* < .01 compared to MM treatment.

FSM = Feng spinal mobilization, MM = Maitland posteroanterior mobilization, SLR = straight leg raise.

### 3.3. Analysis of symptoms before and after MM treatments in 2 weeks

After the MM treatments, in 2 weeks the patient subjective symptoms all improved. The VAS score was significantly reduced from 3.95 ± 1.84 to 2.90 ± 1.82 (*P* < .01), the ODI value was significantly reduced from 26.94%±14.76% to 20.31%±11.19% (*P* < .01), and the PHQ-9 score was significantly reduced from 4.80%±5.26% to 3.13%±3.37% (*P* < .01)(Table 2, Fig. 2). However, no significant difference in all objective outcome measurements (mSchober test, extension ROM, left and right SLR height, flexion ROM, and side flexion ROM) was observed after MM treatment (*P* > .05) (Tables 3 and 4, Figs. 3 and 4).

### 3.4. Comparison of the improvement between FSM and MM treatments

Immediately after the 2 weeks of treatments, compared to the MM treatment, FSM treatment showed a much more significant improvement in VAS score (FSM 2.33 ± 1.87 vs MM 2.90 ± 1.82, *P* < .01), extension ROM (FSM 6.87 ± 3.68 vs MM 5.85 ± 4.35, *P* < .01), left SLR height (FSM 82.30 ± 5.78 vs MM 81.47 ± 5.99, *P* < .05), and right SLR height (FSM 82.04 ± 5.79 vs MM 80.20 ± 6.43, *P* < .05). However, no statistically significant differences were found between FSM and MM treatment for ODI score, PHQ-9, the mSchober test, flexion ROM, and side flexion ROM (*P* > .05) (Tables 2 and 3).

**Figure 5.**
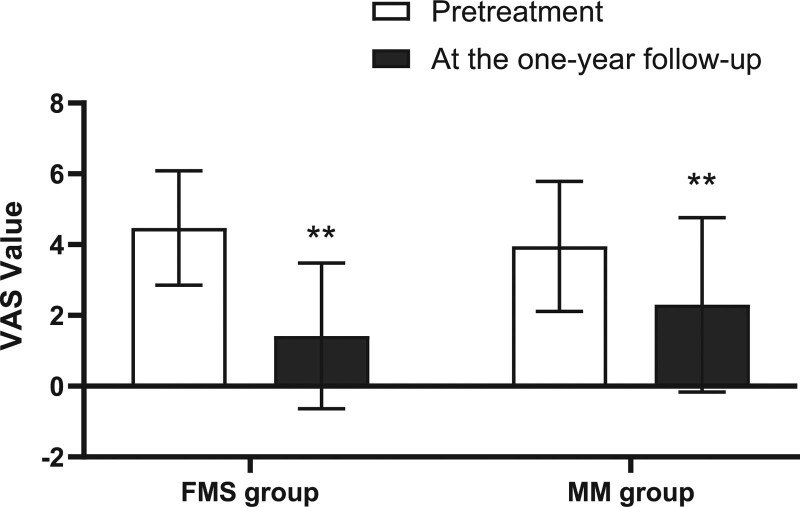
Comparison of VAS between 2 groups at the 1-year follow-up. VAS = visual analogue pain scale, FSM = Feng spinal mobilization, MM = Maitland posteroanterior mobilization. Values are presented as mean S.D. *P < .05, **P < .01 compared to pretreatment, #*P* < .05, ## *P* < .01 compared to MM group.

At the 1-year follow-up visit, the VAS score was significantly reduced from 4.47 ± 1.62 to 1.42 ± 2.06 in the FMS group compared to pretreatment (*P* < .01). In the MM group, the VAS score was also significantly reduced from 4.47 ± 1.62 to 1.42 ± 2.06 when compared to pretreatment (*P* < .01). Although the VAS values decreased more in the FMS group, there was no significant difference in the comparison of changes between the 2 groups (Table 5, Fig. 5).

**Table 5:** Comparison of visual analogue pain scale between 2 groups at the 1-year follow-up.

Time	VAS in FSM group (n = 43)	VAS in MM group (n = 40)	*P* value
Pre treatment	4.47 ± 1.62	3.95 ± 1.84	.179
At the 1-year follow-up	1.42 ± 2.06**	2.30 ± 2.46**	.636
*P* value	.000001	.000462	

Values are presented as mean ± S.D. *P < .05, **P < .01 compared to pretreatment, #*P* < .05, ## *P* < .01 compared to MM treatment.

FSM = Feng spinal mobilization, MM = Maitland posteroanterior mobilization, VAS = visual analogue pain scale.

## 4. Discussion

This study aimed to determine whether the FSM treatment is more effective than the MM treatment in reducing pain, improving back pain-related functional disabilities, psychological states, and objective measurements for patients with CNLBP. Our results demonstrated that FSM treatment significantly improved ROM and bilateral SLR heights in CNLBP patients after treatments, but MM treatment did not. Although both treatments significantly relieved the subjective symptoms, the FSM treatment exhibited more significant improvements in changes of VAS score, extension ROM, and bilateral SLR heights compared to the MM treatment. These findings indicated better efficacy of the FSM treatment than that of MM in CNLBP patients in a brief time, providing an important insight into the clinical practice of management of these CNLBP patients.

The findings that FSM and MM treatments significantly improved subjective symptoms in CNLBP patients are in agreement with previous studies that mobilization can decrease pain, increase function.^[9]^ In addition, we added the psychological evaluation based on the PHQ-9 questionnaire, our results proved the efficacy of the FSM treatment for CNLBP patients both in physiological and psychological states.

In this study, FSM treatment significantly improved the value of mSchober test, lumbar extension ROM, bilateral SLR height in CNLBP patients, and while MM treatment did not. In previous studies, significant improvements in subjective symptoms, lumbar mobility, and SLR height were also observed in patients with lumbar disc herniation (LDH) after FSM treatment.^[31]^ The author explained that the FSM reduced the tension of the nerve sheath and relieved the nerve root compression, thus resulting in improvement.^[31]^ However, in our study, the subjects are patients with CNLBP, not LDH. So, the improvement of the VAS, ROM, SLR height may have both neurophysiological and biomechanical reasons. Research proved that lumbar mobilizations could improve the hamstring tissue extensibility and reduce the tension, stiffness, and electromyographic activity during active movement of the hamstring and erector spinae.^[29]^ Additionally, mobilizations have been found to activate the periaqueductal gray and inhibit temporal summation, which decreases the excitability of dorsal horn cells.^[32]^ So the transient attenuation of alpha motor neuron excitability induced by FMS may decrease muscle tension of involved segments and improve muscle spasm, which may result in gains in joint ROM and SLR height. According to Professor Feng, FSM manipulation is believed to correct vertebral displacement and joint dislocation, and thereby improving joint function and ROM.^[14]^

Previous studies on the effect of MM on ROM have been controversial.^[9]^ The results of MM treatment did not show significant effects on ROM in this study that are consistent with those of Allison and Goodsell.^[33,34]^ These results are in contrast to those of Lee and Ali.^[35,36]^ These discrepancies may be explained by the differences in the Initial ROM measurements among the patients. Stamos-Papastamos found that those who have more initial ROM limitations seem to gain more improvement after treatment.^[37]^ Ali found ROM improvement after the MM treatment,^[35]^ the subjects in their study all had initial ROM limitations, and although the patients with CNLBP in our study did not all display initial stiffness. Furthermore, variations in the mobilization time may also help explain the conflicting results. The mobilization protocol differs from 2 minutes to 9 minutes, which resulted in different outcomes.^[34,35,38]^ Ali found significant increases in lumbar ROM after MM treatment lasting 6 minutes.^[35]^ The 3-minute protocol in our study may not be long enough to cause a significant change. Though no significant objective outcomes were observed, we can still note a trend that some measurements mildly improved when compared with the initial results.

Our results found that the FMS treatment provided better improvements in VAS scores, lumbar extension ROM, and SLR height than the MM treatment. Feng found that patients whose lower back pain had a longer duration always are seen to have lumbar curve kyphosis, spinal scoliosis, and muscle spasms with more serious limitations of lumbar extension.^[39]^ The FMS can improve the VAS scores and the lumbar curve. Zhang found that after the FMS, a patient with LDH had a more symmetrical body shape and improved spinal mobility.^[16]^ Our study confirmed that FSM could remarkably improve the lumbar extension ROM and correct the lumbar joint function. These results may be related to biomechanical reasons because when doing the extension movement, the muscle and ligament around the joint are loose. The joint then glides down, and the pressure in the joint increases, so the extension ROM increase means the joint dislocation is improved. The better result of SLR may be due to the increase in posterior chain neurodynamics and hamstring extensibility.^[40]^ Our study also found a significant improvement in overall pain in the FMS group at the 1-year follow-up. This result is probably because of the correction of misaligned joints by FMS manipulation, which facilitated the recovery of pain.

## 5. Limitations

There are a few limitations in this study. Though there were no significant differences between the 2 groups, the patients with FSM started with a higher mean VAS value and therefore it is normal to expect a higher improvement than patients treated with MM; besides, the preintervention sporting or fitness habits of the patient sample were not evaluated. Hence future studies are expected to consider these parameters in CNLBP patients. What more, a large sample and multicenter study can be conducted, more observables such as gait tests, ultrasound evaluations, and surface EMG tests can be added for further investigating the mechanism details.

## 6. Conclusion

In summary, our study demonstrated that FSM treatment significantly improved CNLBP patients Modified Schober test, extension ROM, and SLR height, while MM treatment did not. Both treatments significantly decreased the values of VAS, ODI, and PHQ-9. Compared to the MM treatment, the FSM treatment showed a more significant improvement in VAS score, range of motion of extension, and SLR of both sides. Our data suggested that FSM treatment can provide better efficacy than MM in CNLBP patients, improving the VAS scores, lumbar extension ROM, and SLR height in a brief time.

## Author contributions

**Conceptualization:** Ying Xie.

**Data curation:** Cheng Gong, Shiyin Dai, Bing Jin.

**Funding acquisition:** Cheng Gong.

**Methodology:** Ying Xie.

**Supervision:** Ying Xie.

**Writing – original draft:** Cheng Gong.
